# Novel Microarrays for Simultaneous Serodiagnosis of Multiple Antiviral Antibodies

**DOI:** 10.1371/journal.pone.0081726

**Published:** 2013-12-18

**Authors:** Ponnurengam Malliappan Sivakumar, Nozomi Moritsugu, Sei Obuse, Takashi Isoshima, Hideo Tashiro, Yoshihiro Ito

**Affiliations:** 1 Nano Medical Engineering Laboratory, RIKEN, Wako, Saitama, Japan; 2 Consonal Biotechnologies Co., Ltd., Funabashi, Chiba, Japan; 3 Emergent Bioengineering Materials Research Team, RIKEN Center for Emergent Matter Science, Wako, Saitama, Japan; Kliniken der Stadt Köln gGmbH, Germany

## Abstract

We developed an automated diagnostic system for the detection of virus-specific immunoglobulin Gs (IgGs) that was based on a microarray platform. We compared efficacies of our automated system with conventional enzyme immunoassays (EIAs). Viruses were immobilized to microarrays using a radical cross-linking reaction that was induced by photo-irradiation. A new photoreactive polymer containing perfluorophenyl azide (PFPA) and poly(ethylene glycol) methacrylate was prepared and coated on plates. Inactivated measles, rubella, mumps, Varicella-Zoster and recombinant Epstein-Barr viruse antigen were added to coated plates, and irradiated with ultraviolet light to facilitate immobilization. Virus-specific IgGs in healthy human sera were assayed using these prepared microarrays and the results obtained compared with those from conventional EIAs. We observed high correlation (0.79–0.96) in the results between the automated microarray technique and EIAs. The microarray-based assay was more rapid, involved less reagents and sample, and was easier to conduct compared with conventional EIA techniques. The automated microarray system was further improved by introducing reagent storage reservoirs inside the chamber, thereby conserving the use of expensive reagents and antibodies. We considered the microarray format to be suitable for rapid and multiple serological diagnoses of viral diseases that could be developed further for clinical applications.

## Introduction

Parallel detection of antibodies with varying specificities has the potential to be a powerful technique in the diagnosis of allergic, autoimmune and infectious diseases. Using conventional immunoassays is time consuming; further, the amount of sample and reagent required limits any high-throughput application of these assays. Therefore, microarrays could be an appropriate substitute for these immunoassays in a clinical setting [1]. Many microarray formats for the detection of antibodies have been developed using various types of disease specific antigens, such as recombinant or tumor-associated antigens to name a few [2]–[10]. Physical adsorption, ionic bonds, or covalent attachment are used for the immobilization of antigens to the microarray surface. Of the immobilization methods employed, covalent immobilization is generally suitable for biomolecules being applied to a limited area because of its stability and efficiency. However, covalent attachment generally requires special functional groups, such as amino or carboxyl groups. Hence, immobilization by photo-irradiation was found to be a suitable alternative and has been used in the preparation of microarray formats by our group and other researchers [11]–[19].

Photo-immobilization methods have several advantages: it is possible to immobilize any organic material to any organic surface, and this is not limited by functional groups; and the random orientation of photo-immobilized probe molecules exposes various sites for interaction with the target molecule. The latter situation enables efficient detection of polyclonal antibodies that contain various epitopes. Therefore, crosslinking via photo-immobilization is considered to be more suitable for antibody detection in serum compared with conventional immobilization methods.

We have previously reported the synthesis and use of a photoreactive polymer with the aid of an azidophenyl-derived non-fouling polymer [13]–[17]. However, azidophenyl-derivatized polymer was not considered to be enough for virus particles. Therefore, in this study to efficiently immobilize virus particles a powerful cross-linker group, perfluorophenyl azide (PFPA) [20], was employed. A new photoreactive polymer was prepared using a non-biofoulant polymer consisting of poly(ethyleneglycol) (PEG) methacrylate and *N*-(2-acrolylaminoethyl)-4-azido-2,3,5,6-tetrafluorobenzamide. PEG is thought to reduce non-specific interactions, and perfluorophenyl azide acts as a photo-crosslinker for immobilization in the presence of ultraviolet (UV) radiation.

## Materials and Methods

### Reagents and sera

PEG methacrylate (350 Da) and bovine serum albumin (BSA) were purchased from Sigma-Aldrich (Milwaukee, WI, USA). A polyclonal affinity-purified horseradish peroxidase (HRP)-labeled goat anti-human IgG antibody was purchased from GE Healthcare (Oxford, UK). The ECL Advance Kit for the detection of HRP was purchased from Amersham Biosciences UK (Little Chalfont, UK). Viruses (Varicella-Zoster, measles, rubella, and mumps viruses) were inactivated by irradiating with UV light as done for clinical analysis kits. The Epstein-Barr virus (EBV) antigen we used was a recombinant p18 viral capsid fused to glutathione S-transferase (GST) which is also employed for clinical analysis. All other reagents were purchased from Wako Pure Chemical Industries (Osaka, Japan). Serum samples from healthy humans were provided from Denka Seiken (Tokyo, Japan) with informed consent provided from individuals. This study and all experiments were approved by the RIKEN ethics committee.

### Preparation of photoreactive PEG

**Figure 1:**
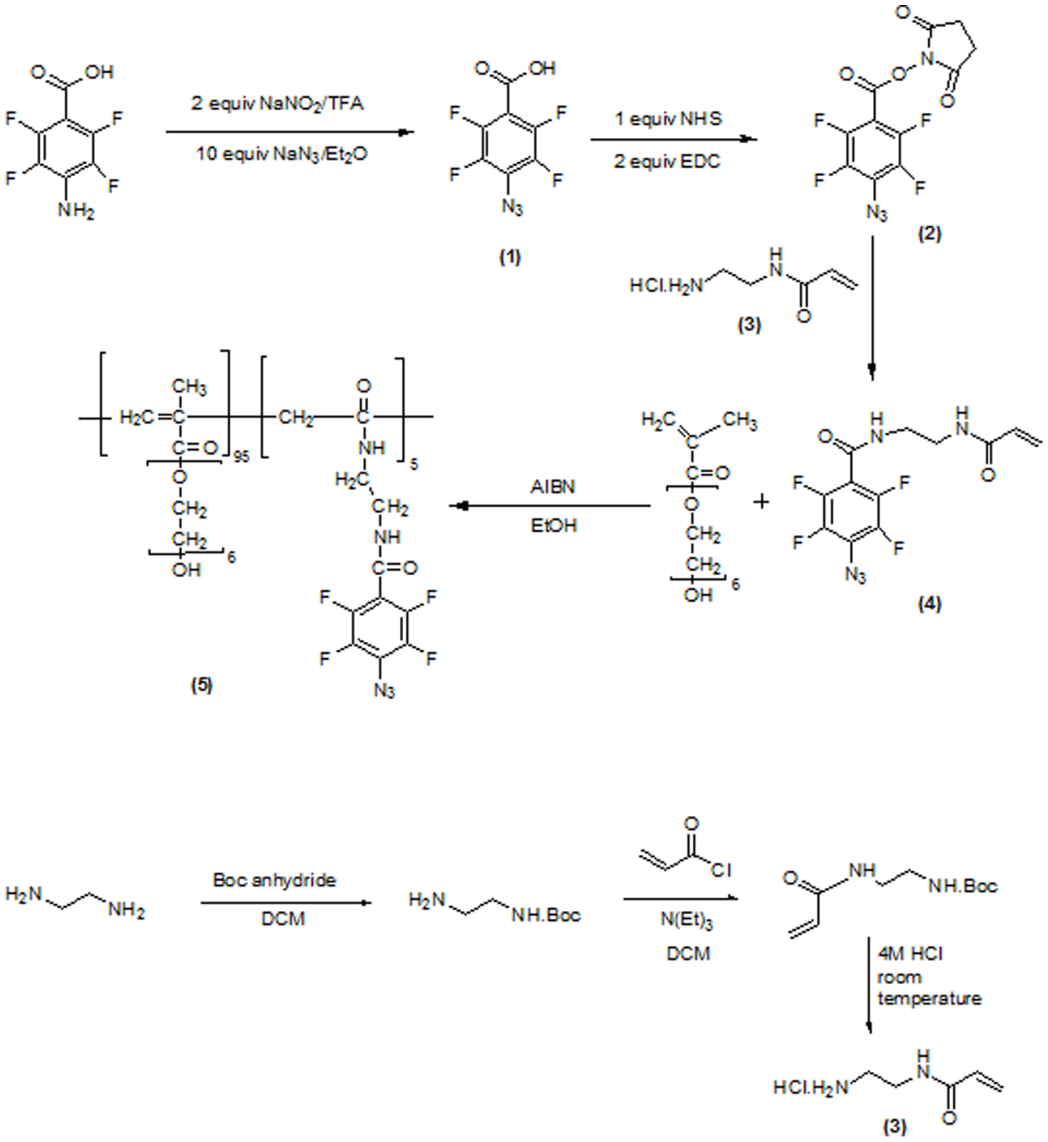
Synthesis scheme for photo-reactive PEG.

A photoreactive monomer **(4)** containing a PFPA moiety was prepared and copolymerized with PEG methacrylate. The synthetic route for the photoreactive monomer **(4)** and photoreactive PEG polymer **(5)** is shown in Figure 1.

#### Synthesis of 4-azido-2,3,5,6-tetrafluorobenzoic acid (1)

We obtained 4-amino-2,3,5,6-tetrafluorobenzoic acid commercially and converted it to 4-azido-2,3,5,6-tetrafluorobenzoic acid **(1)** by a diazotization reaction. We dissolved 4-amino-2,3,5,6-tetrafluorobenzoic acid (2.0 g, 9.57 mmol) in trifluoroacetic acid (50 mL). Diazotization was conducted by the addition of sodium nitrite (1.32 g, 19.1 mmol), and the mixture was stirred for 1 h at 0°C in the dark. Sodium azide (6.22 g, 95.7 mmol) was slowly added to the reaction mixture over 5 min and 40 mL of diethyl ether immediately added, and the reaction left for a further 2 h. Once the reaction was complete, the mixture was quenched with water and the product isolated by diethyl ether extraction, followed by a brine wash. The organic layer was dried over magnesium sulfate, filtered, and concentrated using a rotary evaporator [21]. The percentage yield was found to be 89%.

#### Synthesis of 2,5-dioxopyrrolidin-1-yl 4-azido-2,3,5,6-tetrafluorobenzoate (2)

We dissolved 4-azido-2,3,5,6-tetrafluorobenzoic acid **(1)** (1 g, 4.25 mmol), N-hydroxysuccinimide (489 mg, 4.25 mmol) and 1-ethyl-3-(3-dimethylaminopropyl)carbodiimide (EDC) (1.629 g, 8.5 mmol) in 15 mL of dichloromethane and allowed the solution to stir at room temperature overnight. The resulting mixture was diluted with water and extracted using ethyl acetate. The organic layer was washed with water and dried over sodium sulfate, filtered, and concentrated using a rotary evaporator [21]. The percentage yield was found to be 79%.

#### Synthesis of *N*-(2-Aminoethyl) acrylamide hydrochloride (3)

We added 40 mmol of Boc anhydride to 100 mL of dichloromethane, which was then added drop-wise to ethylenediamine (420 mmol of ethylenediamine in 250 mL of dichloromethane) under a nitrogen atmosphere at 0°C. The reaction was allowed to stir overnight at room temperature. The organic phase was isolated and washed with sodium bicarbonate and water. The resultant solution was dried over anhydrous magnesium sulfate, filtered and concentrated using a rotary evaporator, to yield the mono Boc-protected diamine. Acryloyl chloride solution (16 mmol of acryloyl chloride in 30 mL of dichloromethane) was added drop-wise to the mono Boc-protected diamine (14 mmol of mono Boc-protected diamine and 14 mmol of triethylamine in 30 mL of dichloromethane) cooled by an ice bath. The reaction was then stirred at room temperature for 4 h. The resulting reaction mixture was filtered, and washed with water and sodium bicarbonate The reaction mixture was washed again with water and the organic layer was dried over anhydrous magnesium sulfate, filtered and concentrated using a rotary evaporator. The resulting mixture was recrystallized in toluene. The yield was found to be more than 70%. The NMR and mass spectra results were found to correspond with those published previously [22]. Mono Boc-protected aminoethyl acrylamide was deprotected to yield aminoethyl acrylamide hydrochloride. Briefly, mono Boc-protected aminoethyl acrylamide was deprotected by reaction with 4 M HCl for 3 h at room temperature. The reaction mixture was filtered using a Buchner funnel and then dried.

#### Synthesis of N-(2-acrolylaminoethyl)-4-azido-2,3,5,6-tetrafluorobenzamide (4)

N-(2-Aminoethyl) acrylamide hydrochloride and N,N-diisopropylethylamine were dissolved in 3 ml of dimethylformamide. 2,5-Dioxopyrrolidin-1-yl 4-azido-2,3,5,6-tetrafluorobenzoate **(2)** that was previously obtained was separately dissolved in 3.5 ml of dimethylformamide. The latter solution was slowly added to the former solution drop-wise and allowed to stir for 24 h at room temperature. The resulting solution was extracted using ethyl acetate. The ethyl acetate layer was washed using water and a brine solution, dried over sodium sulfate, filtered and evaporated under reduced pressure. The percentage yield was found to be 66%. Table 1 shows the ^19^F-NMR shifts and HR-mass analysis for compounds **(1), (2)** and **(4)**.

**Table 1:** ^19^F NMR shifts and high resolution mass analysis of synthesized compounds.

Compound	^19^F NMR shifts		High Resolution Mass data (M+Na)^+^	
	F_a_	F_b_	Theoretical	Experimental
4-amino-2,3,5,6-tetrafluorobenzoic acid	−162.5	−139.1		
4-azido-2,3,5,6-tetrafluorobenzoic acid **(1)**	−151.0	−137.2	257.9897	257.9896
2,5-dioxopyrrolidin-1-yl 4-azido-2,3,5,6-tetrafluorobenzoate **(2)**	−151.8	−134.2	355.0061	355.0063
N-(2-acrolylaminoethyl)-4-azido-2,3,5,6-tetrafluorobenzamide **(4)**	−150.8	−141.3	354.0585	354.0589

The position of two sets of fluorine atoms (F_a_ and F_b_) in 4-amino-2,3,5,6-tetrafluorobenzoic acid is shown in Figure S1.

#### Synthesis of the photo-reactive PEG polymer (5)

Polymerization was conducted as previously reported [23]. PEG methacrylate (0.68 g, 1.88 mmol) was added to *N*-(2-acrolylaminoethyl)-4-azido-2,3,5,6-tetrafluorobenzamide **(4)** (33.2 mg, 0.1 mmol), with azobisisobutyronitrile (5.6 mg, 0.03 mmol) also added and dissolved in 10 mL of ethanol. Argon gas was used to purge the solution for 20 min and the reaction mixture placed in an oil bath (60°C, 18–24 h). The resultant solution was evaporated to remove ethanol, and dialysis conducted for 1 day to purify the polymer which was obtained in 76% yield. This polymer is referred to as photo-reactive PEG, and was characterized using gel permeation chromatography (GPC), The molecular weight was determined by GPC using a TSKgel G3000SWXL column (Tosoh, Tokyo Japan) at an absorbance of 270 nm. UV absorption spectra of the purified polymer were measured using a V-550 spectrophotometer (Jasco, Tokyo, Japan)

### Photo-immobilization of viruses

**Figure 2:**
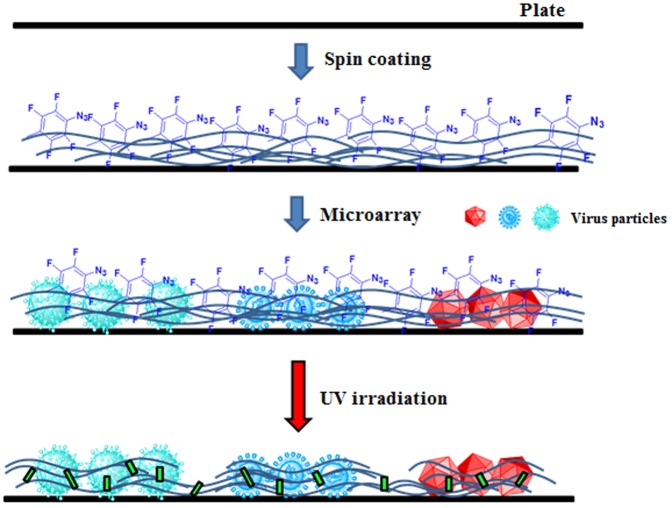
Preparation of the photo-immobilized virus microarray.

The process of virus photo-immobilization is shown in Figure 2. An aqueous solution of 5% photo-reactive PEG was prepared and spin-coated onto a glass plate. Spin-coating was performed under a two-step rotation condition (500 rpm, 5 s and 5000 rpm, 8 s). The virus-containing solutions (1 mg/mL) were prepared using deionized water (50 nL), microspotted onto a plate, and allowed to dry. The microarray plate was irradiated using an UV lamp (300–400 nm; Nippo Electric Co. Ltd FL15BLB, 1.6 mW/cm^2^) for 7 min. The virus-immobilized plates were washed with phosphate-buffered saline (PBS) containing 0.1% Tween-20 (washing buffer) and stored at −20°C until required.

### Thickness measurement

The thickness of the photo-reactive PEG on the coated glass plates was measured with a spectroscopic ellipsometer M-2000DI (J.A. Woollam Co., Nebraska, USA) between 250–1650 nm and at three different incident angles (40°, 45° and 50°). Data were analyzed using the Cauchy model for refractive index dispersion, and the film thickness and refractive index spectrum were obtained by theoretical fitting.

### Surface observation

The surface of the micro spots following viral immobilization were coated with platinum by an ion-coater, then observed using a scanning electron microscope (SEM; JSM6330F, Joel Ltd., Akishima, Japan).

### EIAs

EIAs were conducted using an IgG-EIA kit against the respective viruses commercially available from Denka Seiken (Measles IgG-EIA [SEIKEN], Mumps IgG-EIA [SEIKEN], Rubella IgG-EIA [SEIKEN], Varicella-Zoster IgG-EIA [SEIKEN], and EB VCA IgG-EIA [SEIKEN] for clinical analysis of IgGs against measles virus, mumps virus, rubella virus, varicella-zoster virus, and EB virus (VCA), respectively). Virus was adsorbed to the surfaces of multi-well plates (96 well MicroWell™ PolySorp® flat bottom plate, Thermo Fisher Scientific Inc., Waltham, MA, USA) and diluted serum samples added. After mixing, the plate was left at room temperature for 1 h. The plate was then washed and HRP-conjugated anti-human-IgG antibody was added. Following this, the plate was washed, substrate solution containing 3,3′,5,5′-tetramethylbenzidine and peroxide was added, and the reaction terminated with sulfonic acid. Absorbance values at 450 nm and 630 nm were determined using an auto plate-reader to determine the 450/630 ratio.

### Automated microarray

Serum samples were diluted (1/100) with PBS, and microarray plates with immobilized viruses were incubated with 100 µL of the diluted serum at room temperature for 8 min with shaking. Plates were washed for 3.5 min with 15 mL of washing buffer. An HRP-conjugated anti-human-IgG antibody (diluted 1∶100 with BSA in PBS) was added to the microarray plates and incubated again with shaking for 4 min at room temperature. Plates were washed to remove the non-adsorbed HRP-conjugated anti-human-IgG antibody, and an ECL Advance Kit (3 s, room temperature) used to detect chemiluminescent signals.

### Automated assay system

**Figure 3:**
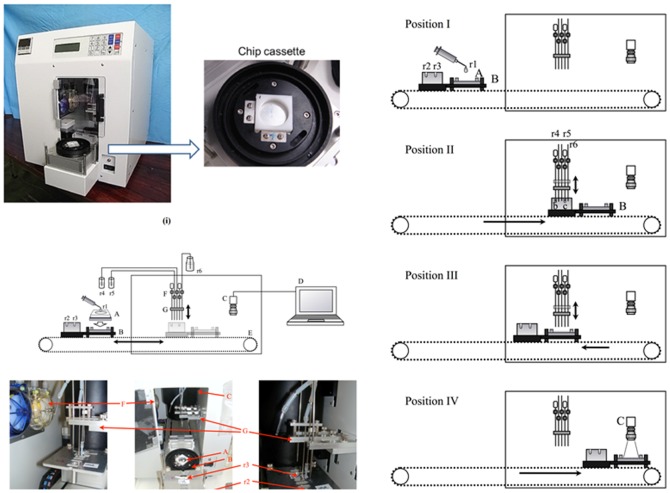
(i) The automated microarray system. (ii) Structure of the automated microarray system. A, protein chip; B, chip and reagent holder; C, digital video camera; D, control and analysis PC; E, belt-drive system; F, pumps; G, needle holder; r1, serum; r2, enzyme-labeled antibody; r3, chemiluminescence reagent; r4, TBS; r5, TBST; r6, liquid waste. (iii) Movements of the automated microarray system. Procedure; Step 1 (preparation for reaction): At Position I, set a protein chip (A) to the holder (B). Inject serum (r1) onto the chip and stir. Inject enzyme-labeled antibody (r2) and chemilumigenic reagent (r3) to the reagent holder (B). Step 2 (wash): The holder moves to Position III, and serum is wasted out. TBS (r4) is injected and wasted, and then TBST (r5) is injected and wasted. Step 3 (reaction): The holder moves to Position II, and r2 is sucked up to the needle. Moves to Position III, r2 is injected on the chip, and stir. Step 4 (wash): The holder moves to Position III, and b is wasted out. TBS (r4) is injected and wasted, and then TBST (r5) is injected and wasted. Step 5 (preparation for imaging): The holder moves to Position II, and r3 is sucked up to the needle. Moves to Position III, and r3 is injected onto the chip. Step 6 (imaging): The holder moves to Position IV and images are taken by the digital video camera (C). Step 7 (completion): The holder moves back to Position I, and the chip is removed.

An automated system consisting of a personal computer, a charge coupled device (CCD) camera (380,000 pixels), a syringe pump, and standard micropipette manufactured valves. These were used to manipulate the flow of samples, HRP-conjugated anti-human-IgG antibody, and ECL reagents (Figure 3). The automated microarray system, and components of the microarray system are shown in Figure 3(i) and Figure 3(ii), respectively.

### Data analysis

Results from the microarrays were compared with those from the EIAs through the calculation of regression coefficients. True negatives (TN), false positives (FP) and false negatives (FN) were categorized; diagnostic sensitivity (DSN) and diagnostic specificity (DSP) were calculated using the following formulae [24]:
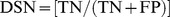


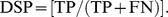



## Results

### Preparation of photo-reactive PEG

**Figure 4:**
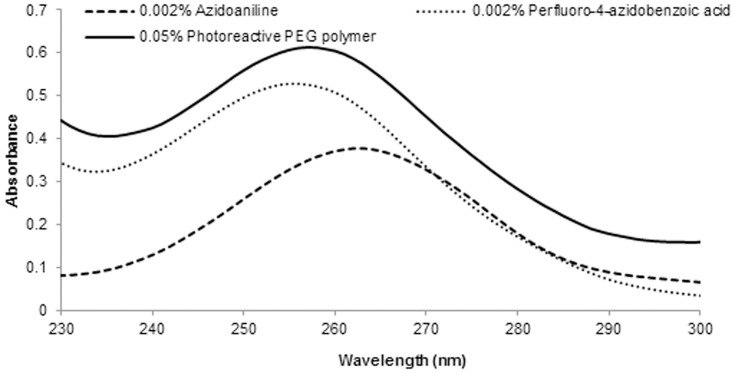
UV spectra of azidoaniline, perfluoro-4-azidobenzoic acid and photo-reactive PEG.

The photo-reactive PEG was successfully prepared and characterized using GPC. The molecular weight of the polymer was approximately 10 kDa. Figure 4 shows the UV spectrum of azidoaniline, perfluoro-4-azidobenzoic acid and photo-reactive PEG. Azidoaniline and perfluoro-4-azidobenzoic acid showed maximum absorption (λ_max_) at 263 and 256 nm, respectively. The photo-reactive PEG had a λ_max_ of 257 nm, while that for 4-azidobenzoic acid was 274 nm (data not shown). Both perfluoroazido-4-benzoic acid and photo-reactive PEG showed a blue shift compared with conventional 4-azidobenzoic acid [25], [26].

### Surface characterization

**Figure 5:**
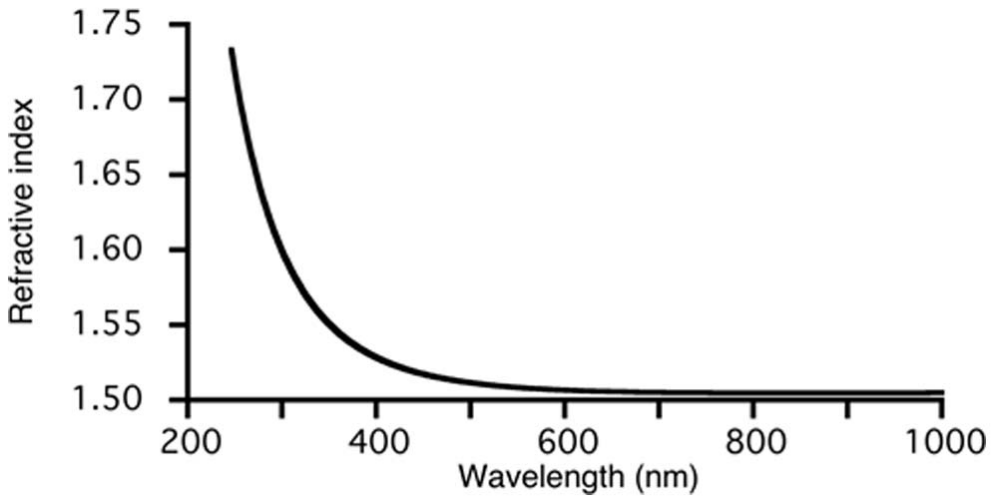
Refractive index of the photo-reactive PEG determined by spectroscopic ellipsometry.

The thickness of the photo-reactive PEG on the glass plate was 38.7±1.3 nm. The refractive index was around 1.50 at longer wavelengths, increasing to 1.7 at shorter wavelengths of 250 nm (Figure 5). For analysis of the ellipsometric data, the Cauchy dispersion model, in which optical absorption was ignored, was used for the photo-reactive PEG layer. This was justified because of the low thickness (38.7 nm), and because the optical absorption band was at wavelengths less than 300 nm. A refractive index of 1.51 at 589 nm is slightly higher than that for PEG (1.46) and PMMA (1.48).

### Photo-immobilization

**Figure 6:**
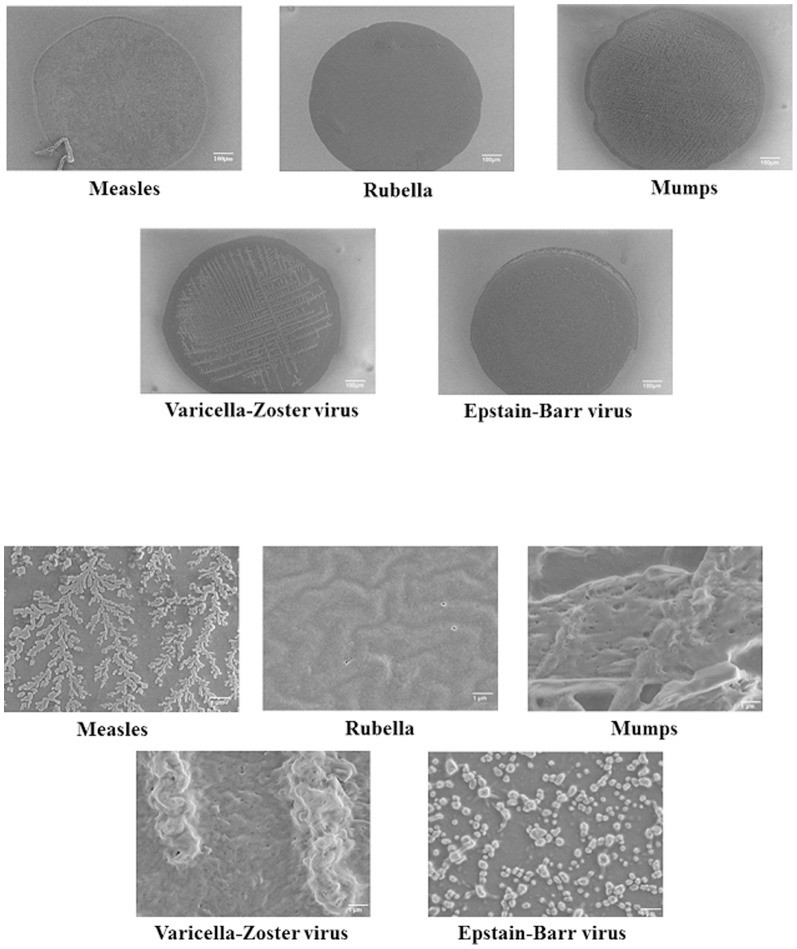
SEM photomicrographs of immobilized viruses.

**Figure 7:**
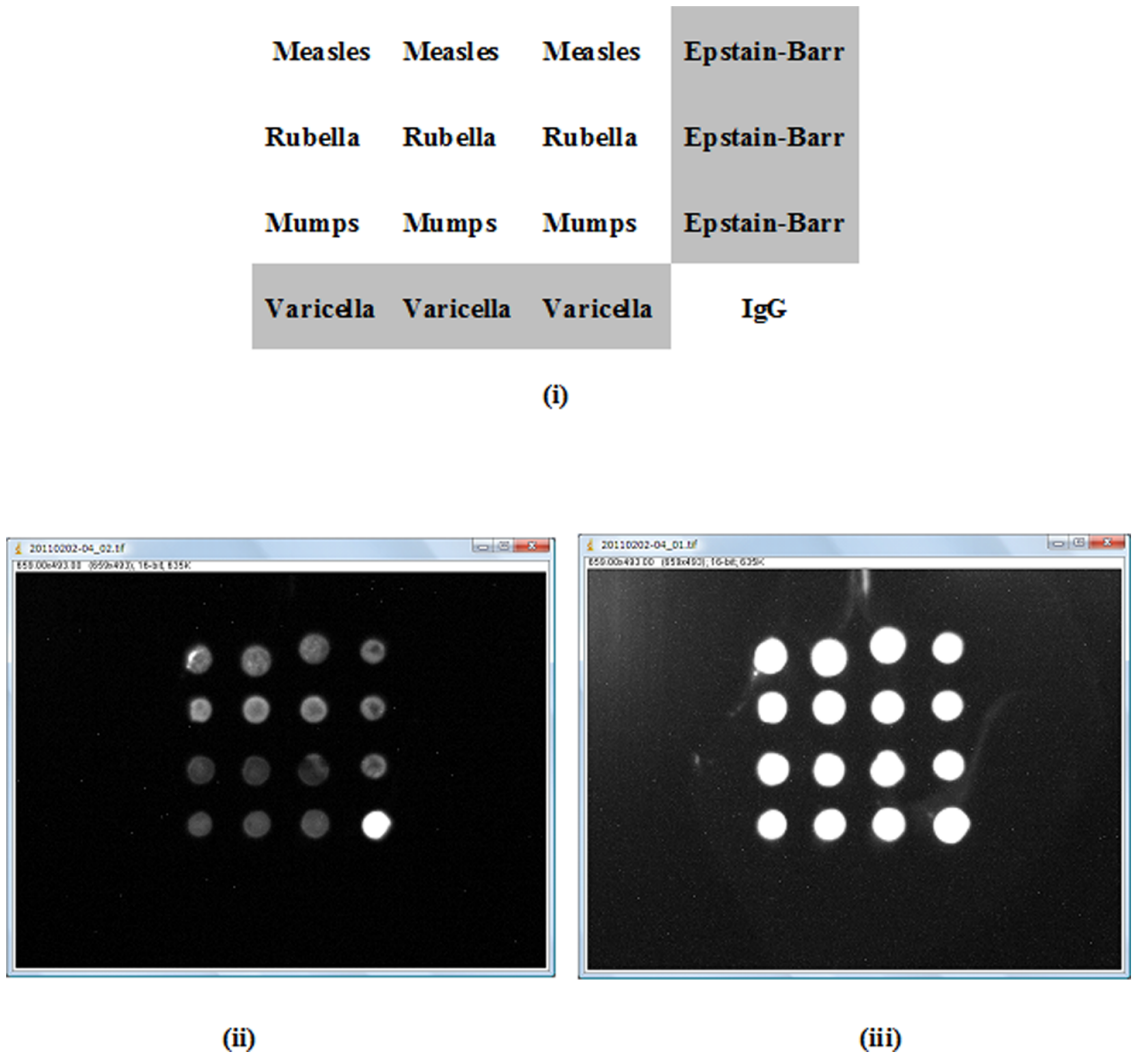
Images of photo-immobilized microarrays following completion of the reaction. (i) Viruses assayed by microarrays (ii) Acquired image following a 5 s exposure. (iii) Acquired image following a 60 s exposure.

The photo-crosslinking reacti`ons were thought to occur between virus and photo-reactive PEG, virus and plate surface, photo-reactive PEG and plate surface, and within PEG chains of the photo-reactive PEG. SEM photomicrographs showed successful photo-immobilization of the various viruses on the microarray plate (Figure 6). Immobilized Virus microspots were stable after washing for 7 min with Tris-buffered saline (TBS) and TBS containing 0.1% Tween-20 (TBST), this is due to the photoimmobilization. We acquired chemiluminescence images of the reaction between the immobilized viruses and serum antibodies (Figure 7).

### Automatic assay system

We automated the microarray assay system such that it was able to analyze a new sample every 20 min. Table 2 shows the flow chart of the automated microarray assay system we designed.

**Table 2:** Flow chart of our automated microarray assay.

	Process	Conditions	Times	Time
1	Primary reaction (immobilized virus and serum IgG)	With shaking	1	8 min
2	TBST washing	With shaking (600 µL each time)	2	3.5 min
3	TBS washing	With shaking (800 µL each time)	13	
4	Second reaction (adsorbed IgG and HRP-conjugated anti-IgG antibody)	With shaking	1	4 min
5	TBST washing	With shaking (600 µL each time)	2	3.5 min
6	TBS washing	With shaking (800 µL each time)	13	
7	Addition of chemical luminescence agents		1	3 s
8	CCD image			5 s and 60 s

Total = approx. 20 min.

### Microarray assays

Results from the microarray assays were compared with those from conventional EIAs. There was a strong correlation between the commercial EIAs and the microarray assays for all viruses. Correlation coefficients were 0.79, 0.96, 0.79, 0.84 and 0.85 for EBV, VZV, mumps, rubella and measles, respectively (Table 3). EIA values of less than 2, 2–4, and greater than 4 were categorized as negative, ambiguous, and positive, respectively. The microarray assay results are provided in Table 4; the DSN was 100% for all microarray results except for measles (94.87%). We found that DSP was absent for all microarray results, except for rubella where it was 100%.

**Table 3:** Statistical equations and correlation coefficients for the comparison between the microarray and EIA results from human serum samples (*n* = 40).

Virus	Equation:		Correlation coefficient
	Chemiluminesence (microarray) = a_*_ absorbance (EIA)+b		
	a	b	
Epstain-Barr virus	184	747	0.79
Varicella-Zoster virus	320	−1313	0.96
Mumps	1509	−101.26	0.79
Rubella	106	−590	0.84
Measles	562	7039	0.85

**Table 4:** Results from various virus microarrays compared with EIA results, along with observed diagnostic specificity (DSP) and diagnostic sensitivity (DSN).

Antibody	No of samples (obtained both Microarray and EIA)	Micorarray results against antibody (compared to EIA)			DSP	DSN
		IgG positive	IgG negative	E		
Epstain-Barr Virus-G positive	40	39	1	0	―	100%
Epstain-Barr Virus-G negative		0	0	0		
Varicella-Zoster virus G positive	36	34	0	1	―	100%
Varicella-Zoster virus G negative		0	0	1		
Mumps G positive	35	21	0	11	―	100%
Mumps G negative		0	0	3		
Rubella G positive	40	34	0	0	100%	100%
Rubella G negative		0	5	1		
Measles G positive	40	37	0	0	―	95%
Measles G negative		2	0	1		

DSP, diagnostic specificity; DSN, diagnostic sensitivity.

## Discussion

Photo-immobilization is considered a suitable alternative to conventional immobilization techniques for biomolecules. Various chemical moieties including arylazides [27], [28], aryldiazirines [29]–[31], benzophenones [32], [33] and nitrobenziles have been used in photo-immobilization studies. The advantage of these compounds is that they can be activated at wavelengths greater than 350 nm. PFPA produces equal C-H insertions to the pentafluorophenyl azide, whereas 2,6-difluorophenyl azide had 17% less C-H insertions in pentafluorophenyl azide [34]. Introduction of electron withdrawing substitutions, such as fluoro and nitro substitutions stabilizes the nitrene and increases photo-immobilization efficiency [34]. PFPAs require less UV irradiation energy compared with phenyl azides, resulting in less damage to the biomolecules [20].

Previous research on photo-reactive PEG has shown it to be a stable, uniform, suitable for miniaturization, and appropriate for photo-immobilization studies of cells and proteins. Because the PEG chains in photo-reactive PEG acts as non-biofoulant surface, it reduces non-specific adsorption [23], [26].

The DSN of all assays was greater than 95%, except in the case of measles; DSP could not be estimated because of a lack of samples. Messazoma et al. [1] and Jaaskelainen et al. [24] reported diverse sensitivities of 50–100% and 62.5–91.8%, respectively, for the detection of antibodies using virus protein microarrays. Jaaskelainen et al. [24] considered that the low sensitivity was due to differences in the antigens employed for EIAs and microarrays, and a reduction in antigenicity caused by preparation, immobilization, or storage of recombinant antigens. In this study, we used the same antigens for EIAs and microarrays. Photo-immobilization of the antigens for both assay types allowed the antigen to maintain its activity.

## Conclusion

Microarrays can be used to perform multiple assays against different viruses with a small volume (<2 µL) of patient serum. Photo-reactive PEG was successfully prepared and characterized then used to photo-immobilize five different viruses on microarray plates. The primary reaction was conducted for 8 min in our automated microarray system, whereas it took 1 h in a conventional EIA. The conventional EIAs also required more reagents than the microarray system. Optimization of parameters such as microspot size, number of viruses that can be immobilized, the diameter between each spot, and assay duration need to be conducted to further improve our system. Using our automated microarray format, only 20 min was required to analyze each sample, and the data were quantitative and reproducible, making it suitable for clinical use.

## Supporting Information

Figure S1 Positions of two sets of Fluorine atoms (F_a_ and F_b_) in 4-amino-2,3,5,6-tetrafluorobenzoic acid.
(PDF)
