# Supplementary figures and images for: Novel Microarrays for Simultaneous Serodiagnosis of Multiple Antiviral Antibodies
Source: PLoS One. 2013 Dec 18;8(12):e81726. doi: 10.1371/journal.pone.0081726 (PMC3867344; doi:10.1371/journal.pone.0081726)

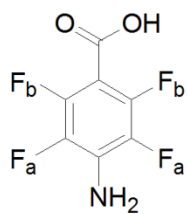

Figure S1      The position of two sets of fluorine atoms (Fa and Fb) in 4-amino-2,3,5,6-tetrafluorobenzoic acid.

Supplement: Figure S1 — Positions of two sets of Fluorine atoms (Fa and Fb) in 4-amino-2,3,5,6-tetrafluorobenzoic acid. (PDF) [file pone.0081726.s001.pdf]
